# Perioperative fluid therapy in adults and children: a narrative review

**DOI:** 10.3389/fmed.2025.1607670

**Published:** 2025-08-01

**Authors:** Juan Victor Lorente, Mónica Hervías Sanz, Javier Ripollés-Melchor, Robert G. Hahn

**Affiliations:** ^1^Department of Anesthesiology and Critical Care, Juan Ramón Jiménez University Hospital, Huelva, Spain; ^2^Fluid Therapy and Haemodynamics Working Group of the Haemostasis, Fluid Therapy and Transfusional Medicine of the Spanish Society of Anesthesiology and Resuscitation (SEDAR), Madrid, Spain; ^3^Department of Anesthesiology and Critical Care, Gregorio Marañón General University Hospital, Madrid, Spain; ^4^Paediatric Anaesthesiology Section, Spanish Society of Anesthesiology and Resuscitation (SEDAR), Madrid, Spain; ^5^Department of Anesthesiology and Critical Care, Infanta Leonor Hospital, Madrid, Spain; ^6^Department of Toxicology, Universidad Complutense de Madrid, Madrid, Spain; ^7^Karolinska Institutet at Danderyds Hospital (KIDS), Stockholm, Sweden

**Keywords:** fluid therapy, crystalloids, colloids, fluid balance, Ringer’s, general anesthesia, pediatric surgery, general surgery

## Abstract

Intravenous fluid administration is an important part of the management of the surgical patient. Fluid can be used to compensate for the normal turnover of fluid and electrolytes (maintenance), to replace losses, to expand the extracellular fluid space to maintain adequate circulation (resuscitation), and to provide nutrition. Too little fluid and too much fluid both increase the number of postoperative complications. Balanced crystalloid solutions, such as buffered Ringer’s, Plasma-Lyte^®^, and Sterofundin^®^, are the most widely used fluids. Isotonic (0.9%) sodium chloride should be reserved for alkalotic and/or hyponatremic patients. Small amounts of these fluids (<500 mL) only expand the plasma volume, while larger volumes distribute to one or two interstitial fluid spaces as well. Filling of the second interstitial space (“third space”) greatly prolongs the half-life of the fluid The indications for colloid fluids are limited but include volume support in major hemorrhage when balanced crystalloids volume become large enough to cause adverse effects (>3 L). Maintenance fluids contain glucose and are indicated during the postoperative period before oral hydration is possible. Glucose might also be provided when awaiting surgery. The choice of replacement fluid is governed by the type of losses that have occurred. The goal of infusion fluids during hemorrhage or serious disease changes over time and might be described in the four phases resuscitation, optimization, stabilization, and de-resuscitation. Nutrition fluids are indicated after 1 week without adequate oral nutrition. Fluid therapy during surgery is performed according to the fluid balance approach (minor surgery), the outcome-oriented approach (intermediate-size surgery), or the goal-directed approach (major surgery). Children tolerate prolonged fasting poorly and preoperative fasting for clear fluids should not exceed 1 h. They have a greater tendency to develop hypoglycemia and hyponatremia than adults and, therefore, isotonic crystalloids that minimize these risks should be used during pediatric surgery. The basal daily need for fluid is children is usually taken according to the “4-2-1” rule to which additions can be made depending on the extent of the surgery. Intravenous fluid administration should be continued during the postoperative phase until enteral hydration is feasible.

## 1 Introduction

Intravenous fluid administration is a common intervention in hospital settings and crucial for ensuring optimal patient outcomes. Proper fluid prescription is essential because ineffective fluid management can increase morbidity and mortality, particularly in the postoperative period (1). Customizing fluid regimens according to the type, amount, and infusion rate allows healthcare professionals reduce the risks associated with improper fluid management (2). Like any drug, fluids must be prescribed with a specific indication, selecting the most appropriate regimen based on the patient’s clinical situation and goals, reassessing the necessity of treatment, and attempting to detect the early onset of possible adverse effects (3).

**Figure 1:**
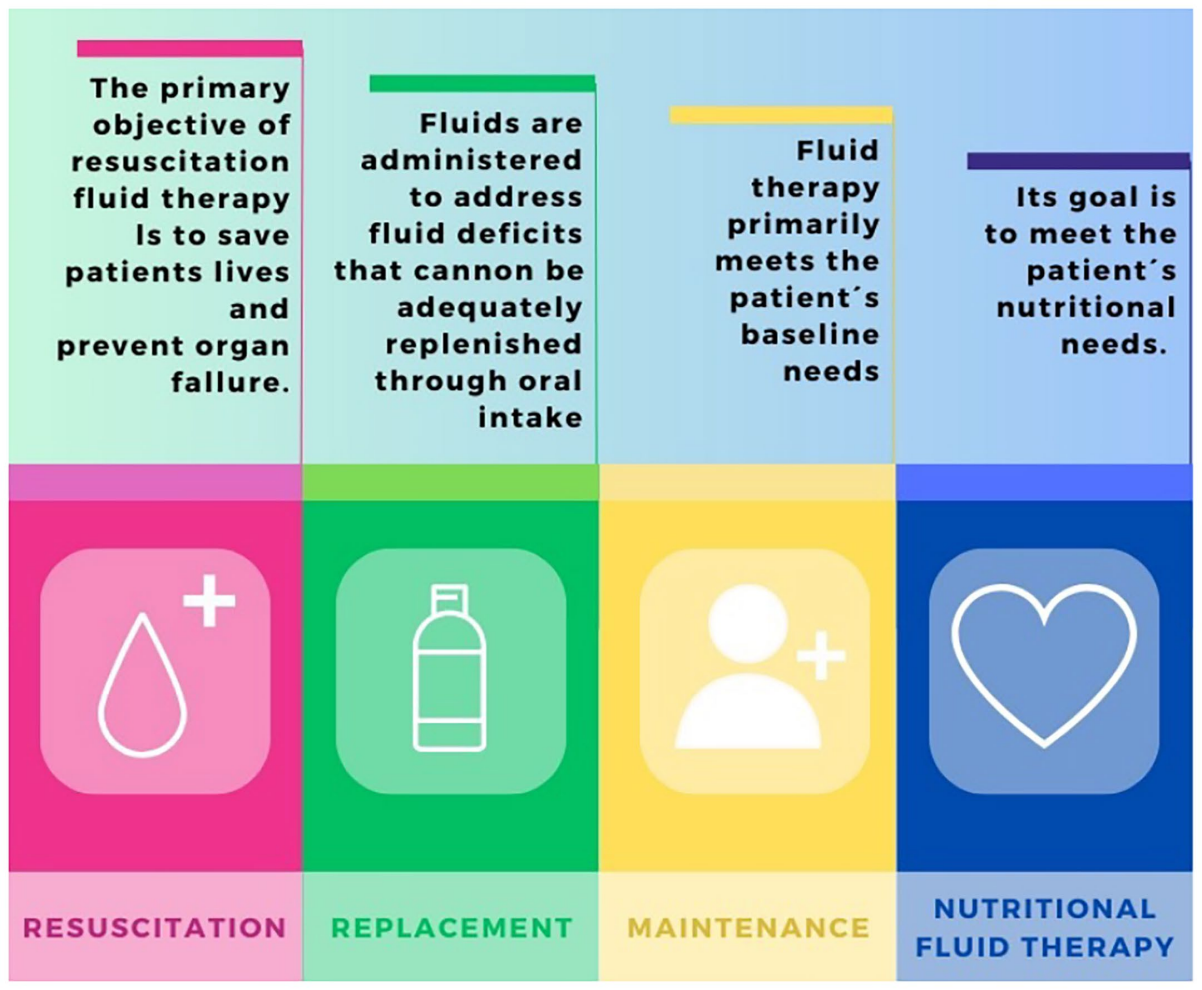
Fluid therapy indications.

There are four main reasons for giving fluid (Figure 1). First, fluids may be used for maintenance therapy, which aims to sustain body fluid volumes and electrolyte levels in patients who are unable to meet their daily needs. Second, replacement therapy compensates for fluid and electrolyte losses, such as vomiting or diarrhea. Third, resuscitation therapy involves expanding the extracellular fluid (ECF) volume, which is needed to maintain adequate circulation during anesthesia and intensive care. Finally, parenteral nutrition serves as a source of fluid administration (4).

These four purposes of providing intravenous fluid are common in perioperative scenarios. Traditionally, research has focused on resuscitative fluid therapy during the intraoperative phase. However, recently published cohort studies reveal that the cumulative impact of fluids given on other indications might surpass that of volumes used for ECF expansion. For example, fluids used as carriers for drugs given by intravenous infusion contribute both to the total volume of administered fluid and to sodium and chloride inputs. In particular, such “fluid creep” is an issue to in the Intensive Care Unit (ICU) (5).

The adverse effects of fluid administration not tailored to patient physiology result from the choice of fluid type, the total volume, and the infusion rate. Fluid overload can cause edema, leading to postoperative complications affecting the lungs, cardiovascular system, gastrointestinal tract, and kidneys (6–8). Conversely, restrictive perioperative fluid therapy (9, 10) and concentrated instead of dilute urine (11) are associated with postoperative acute kidney injury, which is an independent predictor of increased postoperative mortality (12, 13).

Despite its influence on postoperative outcomes, considerable variability remains in intraoperative fluid volume administration among patients with similar comorbidities undergoing comparable surgeries (14), which may further disturb the internal environment, leading to clinically significant complications. Observational studies indicate that a substantial proportion of fluids administered is still isotonic (0.9%) saline rather than fluids that are better balanced regarding their electrolyte content, even in high-risk surgical patients with high comorbidities (15, 16).

This article reviews perioperative fluid therapy in both adult and pediatric populations. The goal was to analyze current evidence and emerging trends to summarize the current understanding of perioperative fluid management, thereby improving patient outcomes and safety in surgical contexts.

## 2 Methods

Given the narrative nature of this review, a comprehensive but non-systematic literature search was carried out to identify relevant publications addressing perioperative fluid therapy in both adult and pediatric surgical populations. The search spanned from January 2000 to May 2025 and was conducted across **PubMed/MEDLINE**, **Scopus**, and **Web of Science** databases. The search strategy combined Medical Subject Headings (MeSH) and free-text terms. Keywords and expressions used included: *“intravenous fluids” “crystalloid solutions,” “colloid solutions,” “intraoperative fluids,” “preoperative fluids,” “preoperative fasting,” “intraoperative fluids,” “postoperative fluid management,” “fluid resuscitation,” “goal-directed fluid therapy,” “maintenance fluids,” “fluid creep,” “volume kinetics,” “fluid overload,” “hypovolemia,” “isotonic solutions,” “hypotonic solutions,” “pediatric anesthesia,” “pediatric fluid therapy,”* and *“perioperative fluid therapy”* using Boolean operators (AND, OR) to combine terms appropriately. Filters were applied to restrict results to human studies and articles published in English or Spanish. Reference lists of key studies and relevant reviews were manually screened to identify additional pertinent sources.

Studies were eligible for inclusion if they were published in English or Spanish and addressed clinical, physiological, or pharmacological aspects of intravenous fluid therapy in the surgical setting. The review considered a wide range of publication types, including systematic reviews and meta-analyses, randomized controlled trials, observational cohort and case–control studies, clinical guidelines, and expert consensus statements. Exclusion criteria comprised studies focusing exclusively on non-surgical or outpatient populations, those with poor methodological quality, and duplicated reports.

Approximately 240 records were initially identified based on the predefined search strategy and selection filters. Given the narrative scope of the review and the aim to capture clinically relevant and methodologically sound studies, a purposive selection process was applied. After title and abstract screening, 108 publications were selected for full-text review and included in the synthesis. The selected literature included 23 systematic reviews and meta-analyses, 19 randomized controlled trials, 31 observational studies, 18 clinical practice guidelines, and 17 expert consensus statements or narrative reviews.

Data were narratively synthesized, focusing on clinical relevance, physiological rationale, and areas of evolving consensus. This integrative approach enabled a structured overview of current evidence applicable to perioperative fluid therapy across diverse surgical populations.

## 3 Fluid kinetics and edema

**Figure 2:**
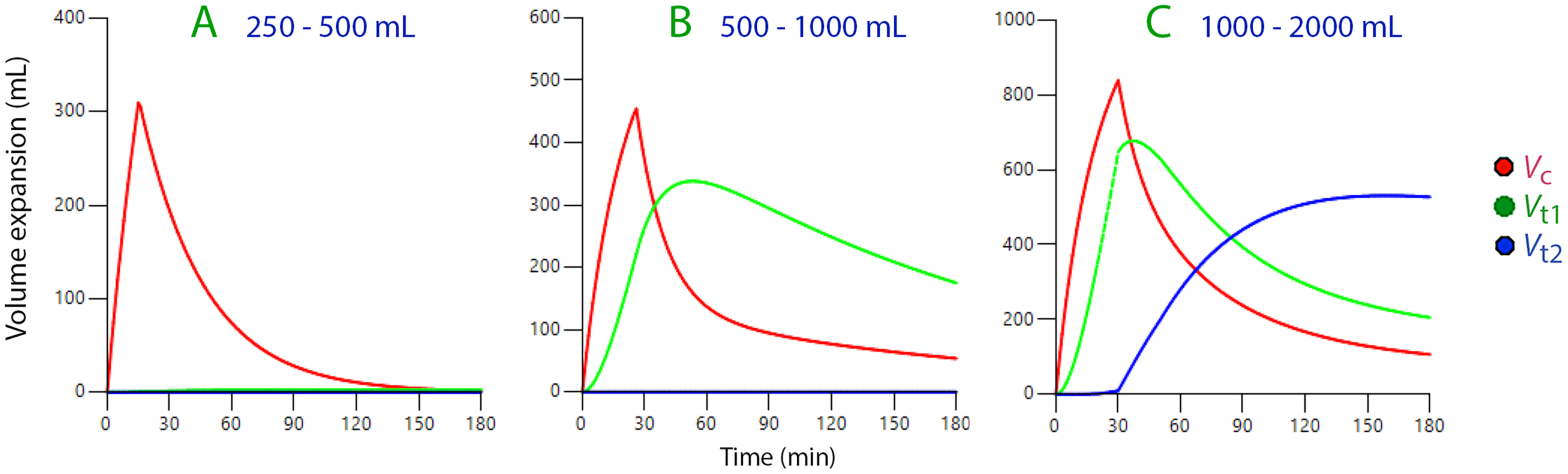
Simulation of the sequential inclusion of up to three body fluid spaces when progressively more crystalloid fluid is infused in humans (**A–C**, infused volumes are shown on top). Note scale changes in scale on the y-axis. Based on data from Hahn (18).

Volume kinetics is a macroscopic method for studying whole-body distribution (17). Crystalloid fluid distributes between three body fluid compartments: a central volume (the plasma), one fast-exchange compartment, and one slow-exchange interstitial compartment (18). These body fluid spaces gradually become filled when more crystalloid fluid is infused. Administration of small amounts (250–500 mL) is distributed only in the plasma space, while larger amounts are distributed to a fast-exchange phase of the interstitial fluid space (*V*_t1_) which has approximately the same size as the plasma volume. Fast infusion (30 min) of >1.3 L increases the interstitial pressure from being lower to be higher than the surrounding air, whereby (probably) the gel phase breaks up and allows accumulation of excess fluid in the slow-exchange interstitial space (*V*_t2_) (19). This sequence is illustrated in Figure 2. Opening of *V*_t2_ might involve the formation of lacuna (small pools of fluid) in the skin (“pitting edema”) but pools may also be found in the heart. The turnover of fluid in *V*_t2_ is very slow which might explain why body weight can increase, and peripheral edema persist, for several days after surgery (6, 20). Excessive amounts of fluid may also accumulate in the *V*_t2_ in inflammatory patients due to a cytokine-mediated decrease in interstitial fluid pressure, which creates a suction effect (19, 21). The importance of fluid accumulation in *V*_t2_ to the outcomes of surgery and intensive care is unclear at present, but many studies demonstrate poorer outcomes when the fluid balance is very positive, which is likely to involve fluid accumulation in *V*_t2_.

Glucose-containing fluids expand the plasma volume to the same degree as Ringer’s, but the effect subsides almost completely 30 min after the infusion ends (20% of the infused volume does *not* remain, as with Ringer’s). The reason for the short-lived volume effect is the intracellular uptake of glucose, which brings along water (22). Moreover, the insulin response to glucose loading causes sodium retention (23).

Adaptation of the hemodynamic system to general anesthesia has three major effects on the kinetics of the crystalloid fluid. The first is impairment of the diuretic response to volume expansion. The inhibition amounts to 85–90% and has been attributed to the anesthesia-induced decrease in arterial pressure (24). The mechanism appears to be that hypotension unloads the baroreceptors, which increases the activity in the renal sympathetic nerves that leads to fluid retention (25). This effect disappears soon after waking from general anesthesia (26). The second adaptation consists in an inhibitory effect of both intravenous and volatile anesthetics on lymphatic pumping. The reduction in the rate parameter for the return of the distributed fluid to the plasma is in the range of 30–50% (27). The third effect is a modest acceleration of the capillary leakage of fluid.

**Figure 3:**
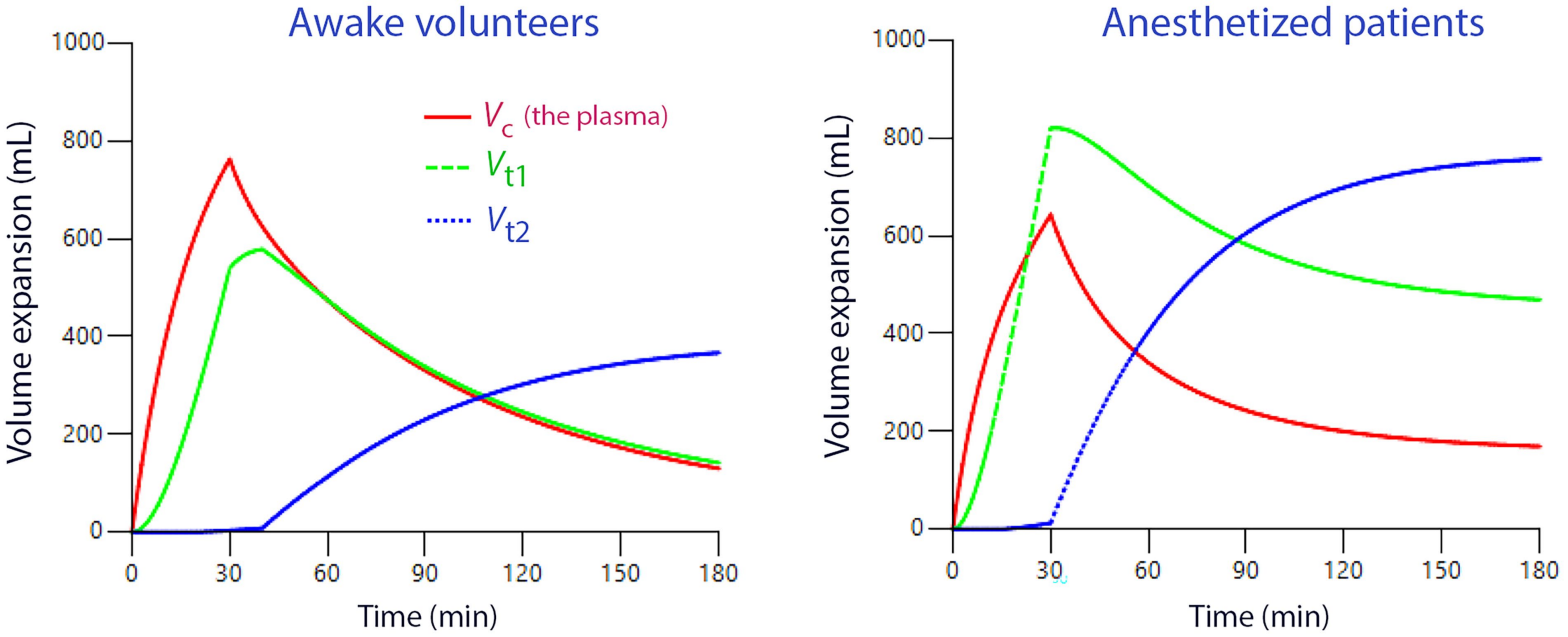
Simulation of the distribution of an infusion of 1.5 L of Ringer’s acetate solution over 30 min in the awake (left subplot) and anesthetized state (right subplot). The greater expansion of *V*_c_ and *V*_t1_ during general anesthesia is due to inhibition of the urine output and the lymphatic flow (kinetic parameters *k*_10_ and *k*_21_) which both act to pressure more fluid into the “third fluid space” (*V*_t2_). Lower infusion rates and fluid volumes < 1 L does not promote “third-spacing” The kinetic data for the analyses were taken from a data bank where 1.0–2.7 L had been infused in 103 volunteers and 1.2–2.8 L had been administered to 54 anesthetized patients undergoing thyroid surgery or open hysterectomy. All fluids were given over 30 min and the two peripheral volumes were assumed to communicate with *V*_c_ (the plasma) in a serial fashion.

Kinetic changes during general anesthesia compared to the awake state are illustrated in Figure 3. Low urine output due to the anesthesia-induced decrease of the arterial pressure retains fluid, which mainly ends up in *V*_t1_ and *V*_t2_. Hence, a moderately severe maldistribution in favor of the extravascular (interstitial) space develops due to the depressive effect of anesthetic drugs on lymphatic pumping, which returns distributed fluid to the plasma.

**Figure 4:**
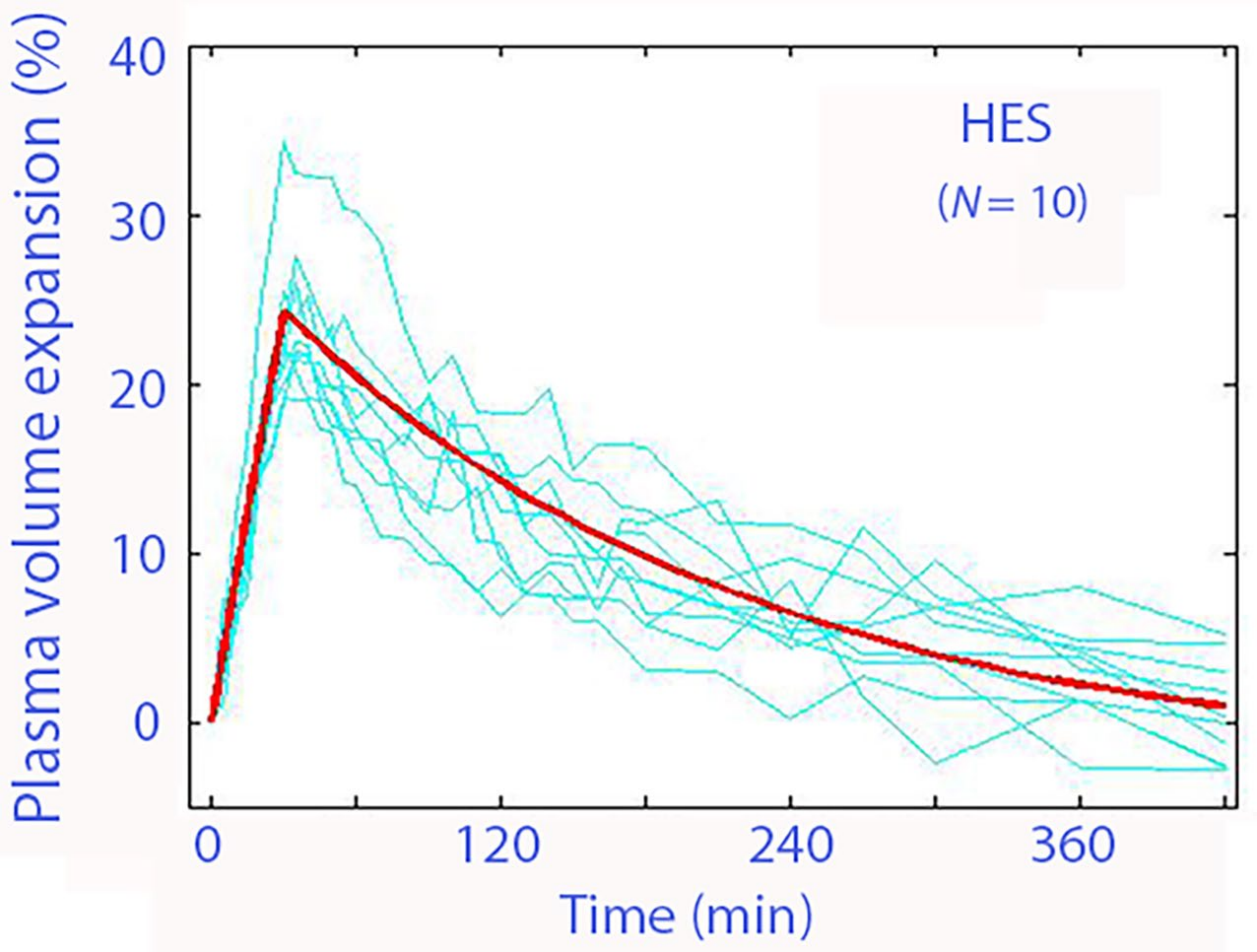
Plasma volume expansion resulting from infusion of 10 mL/kg of hydroxyethyl starch 130/0.4 (Voluven^®^) over 30 min in volunteers. Thin lines are individual experiments, and the thick line is the modeled average curve. From Crit Care 2013; 17: R104.

Colloid fluids do not share the context-dependent kinetics with crystalloid fluids. The plasma volume expansion induced by iso-oncotic colloids subsides slowly over several hours, and no obvious distribution function is observed (Figure 4). The predictable plasma volume expansion is an advantage when repeated fluid boluses are given as part of “goal-directed fluid therapy.” The plasma volume expansion decreases in parallel with the capillary leakage of the administered macromolecules but is followed by urinary excretion of a similar proportion, which results in slight dehydration (28). Hyper-oncotic fluids show a different picture, which is best studied for 20% albumin. This solution attracts water from the interstitium whereby the urine output increases, and the plasma volume expands by twice the infused volume (29, 30).

Increased capillary leakage of intravascular protein and infused colloid molecules has been suggested to occur when the endothelial glycocalyx layer is damaged by surgery, infusion fluid, and inflammation (31–33). Measurements of capillary leakage in humans do not yet clearly support this theory, which originates from laboratory studies of primitive animals (20, 34, 35).

## 4 Intravenous fluids in the perioperative setting

**Figure 5:**
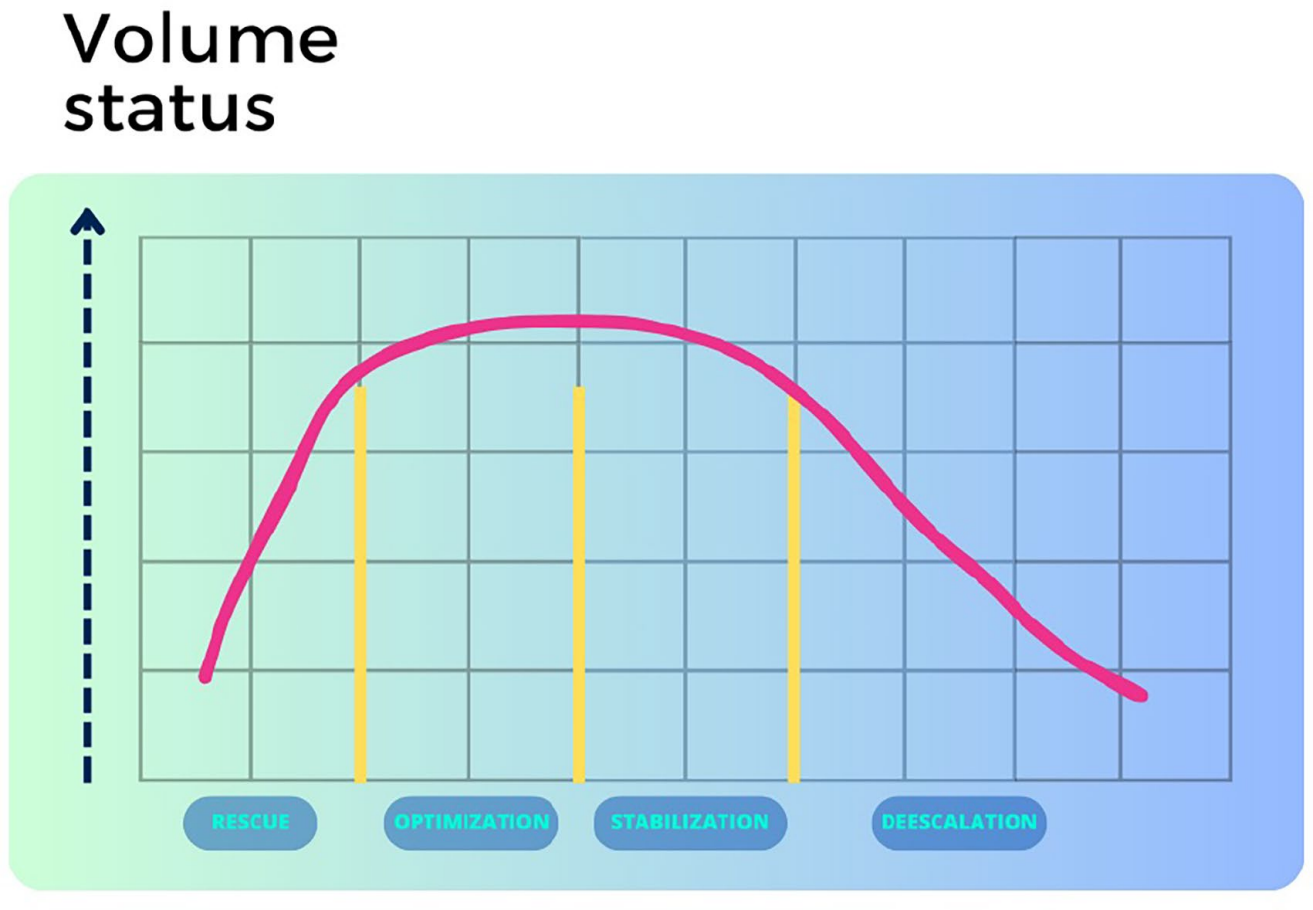
Patient’s volume status at different phases.

The *perioperative period* is the time immediately before, during, and after a surgical procedure. Fluid administration in the clinical context is complex, particularly during the perioperative period, owing to the dynamic nature of human physiology in the surgical situation. The primary choice of fluid is a balanced electrolyte solution, such as lactated or acetated Ringer’s solution. Importantly, the volume status of a surgical patient must be frequently adapted to cope with evolving clinical changes (1) (Figure 5).

In cases where the patient experiences active or recent hemorrhage undergoing emergency surgery or faces a septic condition, the commencement of fluid therapy typically involves an initial *resuscitation phase*. During this critical period, which may involve a state of shock, the primary objective is to quickly restore the equilibrium between oxygen supply and demand (36).

The *optimization phase* aims to sustain normovolemia, which is a goal maintained throughout the intraoperative period. Although hypovolemia may not immediately result in hypoperfusion, research has linked this to adverse outcomes in patients undergoing surgery (37). This phase involves adhering to a goal-directed fluid treatment algorithm as guided by invasive monitoring of central hemodynamics. Conventional non-invasive parameters, such as heart rate, arterial pressure, and urine output, often prove unreliable and react late to hypovolemia (38).

The *stabilization phase* differs from the previous ones. Treatment focuses on organ support when the patient has reached a stable steady state. During this phase, which usually corresponds to the postoperative period after major elective surgery, fluid therapy provides continuous maintenance volume support to compensate for normal fluid and electrolyte losses. The term “de-resuscitation,” proposed in 2012 and officially coined in 2014, specifically refers to late goal-directed fluid withdrawal and late conservative fluid management (39). Delayed goal-directed fluid removal employs an active approach using diuretics and renal replacement therapy to achieve negative fluid balance. In contrast, delayed conservative fluid management adopts a more moderate strategy after the initial treatment to prevent or reverse fluid overload (40). Withdrawal of fluid from an overhydrated patient takes time and might involve hypovolemic and hypotensive episodes if rushed. An explanation for these events might be the strong dominance of fluid accumulation in the “third space” (*V*_t2_) which becomes apparent within a few hours after the resuscitation phase ends (Figure 3). Accumulation of fluid in this space might prolong the half-life of the infused fluid by a factor of 25 as compared to the half-life of 30-50 min that is found when small amounts are infused (18).

### 4.1 Maintenance fluids

The aim of maintenance fluids is to provide water, electrolytes, and glucose to patients who cannot meet these requirements via enteral means during the perioperative phase (41). Consequently, the composition of maintenance fluids must be aligned with the specific indications for their prescription, requiring careful consideration and caution. Abrupt changes in the patient’s normal intake of water and electrolytes should be avoided (3).

#### 4.1.1 Preoperative fluid management

Intravenous maintenance fluid might be provided to improve well-being throughout the preoperative phase, as well as in patients awaiting surgery, because patients need to be in the fasting state when anesthesia is induced. However, current guidelines do not recommend overnight fasting but allow ingestion of solids up to 6 h and fluids up to 2 h before surgery (42). Adherence to these guidelines does not adversely impact patients’ fluid status or responsiveness to fluid therapy, as assessed by passive leg elevation immediately before surgery (43).

Surgery initiates a series of stress physiological processes in the body, inducing transient insulin resistance, which is related to a reduction in the efficacy of insulin to exert its normal anabolic actions. Postoperative insulin resistance has been linked to nausea and vomiting (44), catabolism (45), impairment of well-being (46) and but it can be limited or prevented by insulin (47), preoperative infusion (48, 49) or oral administration of a carbohydrate-rich drink, primarily composed of maltodextrins (50, 51). However, later studies by our group, as well as well as others, do not support that oral carbohydrates reduce insulin resistance, which makes this effect controversial (52, 53). Interestingly, insulin resistance may also have benefits, such as lower risk for arterial hypotension (54). Nevertheless, preoperative consumption of a carbohydrate-rich drink the night before surgery and up to 2 h preoperatively, improves postoperative well-being without increasing gastric volume or aspiration risks (55).

Routine use of systematic mechanical bowel preparation (MBP) before surgery should be discouraged, with limited evidence supporting its necessity except for specific cases of rectal surgery anticipating a protective stoma (56). MBP dehydrates the patient and, if used, should be compensated by fluid administration.

Given the reduced need for MBP and shortened fasting periods, prescribing crystalloid fluids prior to elective surgery is usually considered physiologically unnecessary (57).

#### 4.1.2 Intraoperative maintenance fluid therapy

The body undergoes a series of inflammatory and hormonal reactions in response to surgical stress (58). In addition to insulin resistance, this includes increased aldosterone secretion and activation of the renin-angiotensin II system, leading to potassium loss and retention of sodium and water (59). Additionally, vasopressin boosts water reabsorption in the kidney’s collecting ducts.

Perioperative fluid loss includes urine production, perspiration through the skin and respiratory tract, wounds, blood loss, and fluid shifting into the interstitium. Intravascular volume losses during surgery also includes proteins from wound exudation. The body counteracts these fluid losses by water produced during metabolism (approximately 300 mL/day). Moreover, insensible sweating decreases due to lowered metabolic rate and ambient temperature during surgery, further affecting the fluid balance (60). Historically, surgery-related evaporation losses have been overestimated, leading to excessive fluid therapy, which can worsen the postoperative outcome.

Balanced electrolyte-based crystalloids are recommended for intraoperative fluid therapy because of their ability to distribute throughout the ECF space (61). The suggested crystalloid dose varies depending on the type of surgery, ranging from to 1–2 mL/kg/h for laparoscopic surgery to 3–5 mL/kg/h for laparotomy and abdominal surgery (37). The term “crystalloid fluid” commonly denotes electrolyte-containing fluids, but solutions based on glucose are also crystalloids. They may be indicated in the postoperative period but only on special indications during ongoing surgery owing to the risk of hyperglycemia-induced infection and osmotic diuresis (22).

#### 4.1.3 Postoperative fluid management

Both the NICE and ERAS Guidelines encourage early oral intake in the postoperative period, whereas intravenous fluids should be restricted (62–65). However, maintenance fluid therapy is warranted in specific circumstances associated with the surgical procedure, the patient’s condition, or inability to comply with enhanced recovery protocols (66). The objective of postoperative maintenance fluid therapy is to ensure an adequate supply of free water and electrolytes to sustain internal balance and provide ample glucose to avert a shift toward anaerobic cellular metabolism (ketosis) when oral intake alone is insufficient to meet daily needs.

Numerous studies have demonstrated that maintenance fluids serve as substantial sources of water, sodium, and chloride in both pediatric and adult patients during their tenure in critical care units (5). The normal fluid losses that need to be compensated primarily consist of evaporation from the airways and the baseline urine output (67). Renal water conservation was initiated when oral ingestion of water was below 1.8 L/24 h, corresponding to a urine output of 1 L (68).

The current recommendation given by the National Institute for Clinical Excellence (NICE) is to provide 25–30 mL/kg of water per 24 h along with 1 mmol/L of sodium, chloride, and potassium as maintenance to adults in hospital; this is usually delivered as a 5% glucose solution that is half-isotonic regarding electrolytes (69). The recommendation represents the minimum requirement, as Europeans usually ingest 5-10 mL/kg more water and twice the amount of sodium (70, 71). Glucose in 1 L of 5% glucose contains only 200 kcal and prevents blunt starvation and muscle wasting but should be infused no faster than 100 mL/h in the postoperative period to avoid hyperglycemia. Another reason for providing glucose is that it allows hydration of the intracellular fluid space, which becomes volume depleted by evaporation losses (22, 70).

A study conducted by Uña et al. investigated fluid therapy practices and found that commonly prescribed regimens often fail to adequately meet patients’ daily fluid requirements. Instead, they tend to provide excessive sodium and chloride while lacking sufficient potassium, calcium, and magnesium (69). These electrolyte imbalances have been associated with poorer outcomes after surgery and are the result of the predominant use of isotonic (0.9%) saline solution combined with 5% or 10% glucose solution (72). Their composition deviates considerably from that of the internal environment, mainly because of their high sodium and chloride content (73).

##### 4.1.3.1 Glucose solutions

**Figure 6:**
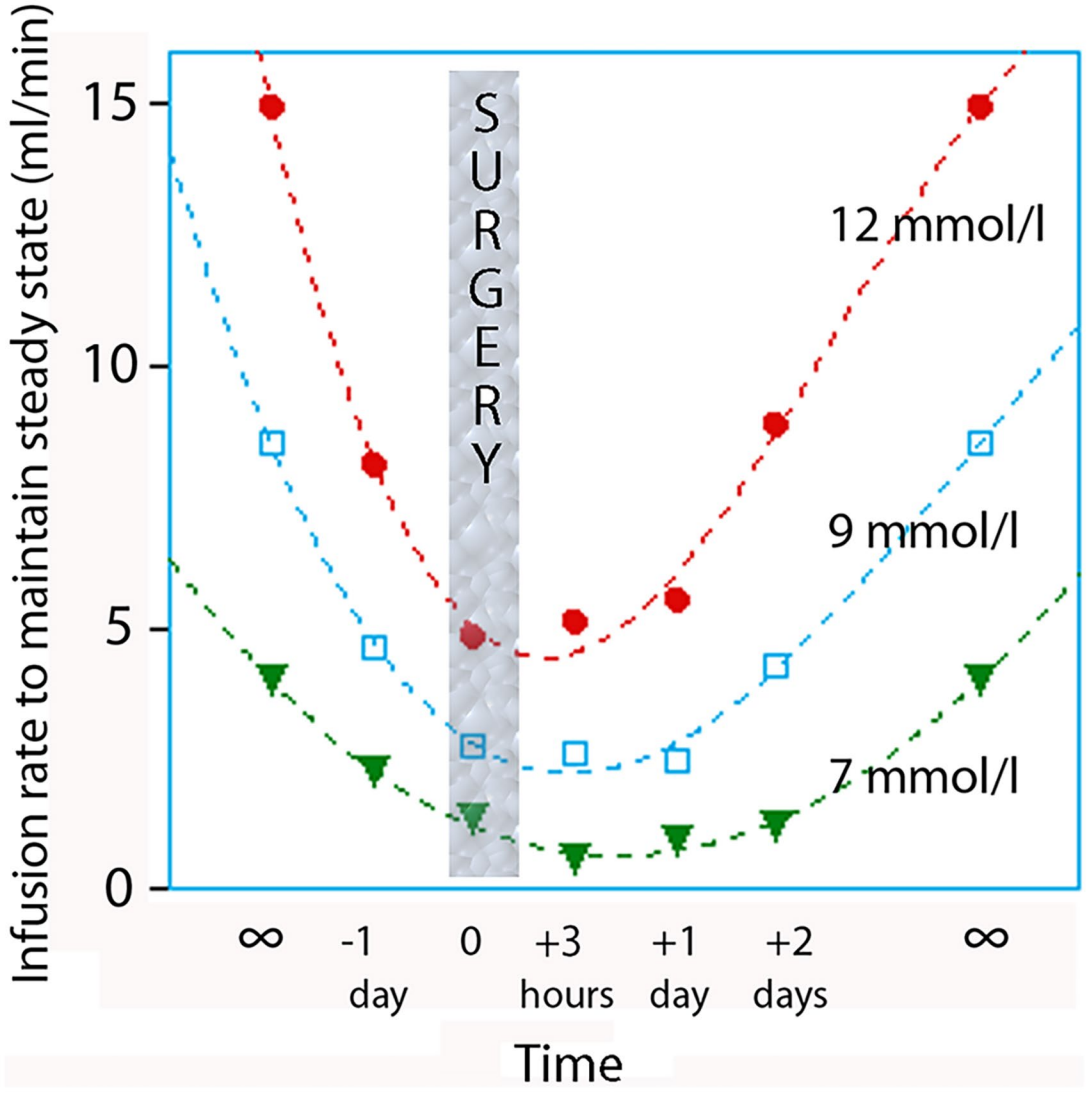
Insulin resistance during the perioperative period. The plot shows the plasma glucose concentration to be expected from various rates of infusion of glucose 5% before, during and after surgery in relatively healthy non-diabetic subjects, standardized to a body weight of 70 kg. Computer simulation based on data from 5 studies of glucose kinetics. Reprinted with permission from Joosten et al. (170).

Glucose (dextrose) solutions are commonly used as maintenance fluids in clinical settings. However, their infusion rates are restricted compared with other crystalloid solutions because of their tendency to elevate plasma glucose levels (74). A notable challenge with glucose solutions is their intravenous administration, which bypasses essential gastrointestinal hormones involved in glucose metabolism content. As a result, intravenous infusions elicit more pronounced hyperglycemic effects than oral infusions, particularly in ICU patients as they frequently develop insulin resistance. It is crucial to prevent plasma glucose concentrations from reaching very high levels (>12 mmol/L) with regular monitoring of plasma glucose levels because of the risk of promoting bacterial growth and inducing osmotic diuresis (75, 76) (Figure 6).

The target for plasma glucose in noncardiac surgery is <10 mmol/L, and no better outcome is achieved by adopting the tighter target of 5.5–7.7 mmol/L (77). The incidence of complications after cardiac surgery was lowest when the plasma glucose varied between 7.8 and 9.4 mmol/L (78).

##### 4.1.3.2 Hypotonic versus isotonic solutions

An alternative approach to postoperative maintenance fluid therapy involves the use of hypotonic fluids rather than isotonic fluids. Isotonic maintenance fluids play a crucial role in averting complications associated with hyponatremia, particularly in children who are notably susceptible to neurological manifestations and hypoglycemia due to electrolyte disturbances. In contrast, isotonic electrolyte solutions are primarily aimed for expanding the ECF volume and provide a large amount of sodium, which places a burden on kidneys. However, advocating isotonic solutions for all hospitalized patients is not warranted, as only a minority face the genuine risk of hyponatremia. This is especially true among adults who typically receive sodium from various sources and undergo regular monitoring of sodium levels during hospitalization.

Nevertheless, research on adults in this area remains limited, with existing studies indicating that administration of isotonic solutions leads to a more positive fluid balance than hypotonic alternatives. Recent findings from a study involving healthy volunteers demonstrated that isotonic solutions (comprising glucose 5% in NaCl 0.9% with added 40 mmol KCl/L) resulted in reduced urine output (and consequently a more positive cumulative fluid balance at 48 h) than hypotonic solutions (specifically Glucion^©^ 5%) (79).

These results were further validated in a study involving 69 critically ill patients following major thoracic surgery (TOPMAST) that utilized the same isotonic and hypotonic fluids. The tonicity of the maintenance fluids played a significant role in influencing perioperative fluid retention, with the isotonic group retaining 4.5 L compared to 3.1 L in the hypotonic group, independent of the administered volume (which was approximately 2.65 L in both groups). At 72 h, isotonic maintenance fluids were associated with an estimated difference of 1,369 mL [95% confidence interval (CI) 601-2137] (80). Furthermore, adopting an isotonic maintenance fluid strategy induced hyperchloremia (>109 mmol/L) in most patients (68.6% vs. 11.8%, *p* < 0.001), whereas hypotonic maintenance fluids led to decreased sodium levels and hyponatremia (<135 mmol/L) in 11.8% vs. 0% (*p* = 0.04) of cases, albeit without any reported clinical effects in adult surgical patients.

Recent research has emphasized that maintenance and replacement fluids constitute the largest portion (24.7%) of the average daily total fluid volume during admission to an ICU, significantly surpassing resuscitation fluids (6.5%). Maintenance fluids also serve as the primary sources of sodium and chloride. Notably, the fluid creep resulting from maintenance and replacement fluids contributes, on average, to one-third of the daily fluid volume (5). During the postoperative period, non-resuscitation fluids have an even greater absolute impact on cumulative fluid balance than resuscitation fluids. Consequently, clinicians should be mindful of inadvertent daily loading of volume, sodium, and chloride when prescribing maintenance fluids.

Crystalloids with a potential use as maintenance fluids are shown in Table 1.

**Table 1:** Crystalloids with a potential use as maintenance fluids.

	NaCl 0.3%	NaCl 0.45%	5% Glucose	10% Glucose	3.3% Glucose	5% Glucose	Benelyte®	Glucion 5%®	Maintelyte®	Plasma
					NaCl 0.9%	NaCl 0.9%				
Na^+^ (mEq/L)	51	77	–	–	154	154	140	54	40	140
K^+^ (mEq/L)			–	–	–	–	4	26	20	4
Ca^2+^ (mEq/L)			–	–	–	–	2	–	–	5
Mg^2+^ (mEq/L)			–	–	–	–	1.5	5	3	2.5
Cl^-^ (mEq/L)	51	77	–	–	154	154	118	55	40	98
Bicarbonate (mEq/L)			–	–	–	–	–	–	23	24
Lactate (mEq/L)			–	–	–	–	–	25	–	–
Acetate (mEq/L)			–	–	–	–	30	–	23	–
Citrate (mEq/L)			–	–	–	–	–	–	–	–
Malate (mEq/L)			–	–	–	–	–	–	–	–
Gluconate (mEq/L)			–	–	–	–		–	–	–
Glucose (g/L)	0	0	50	100	33	50	10	50	50	–
SID *in vivo* (mEq/L)	0	0	0	0	0	30	0	30	23	42
Osmolarity (mOsm/L)	102	54	278		585		351	170	402	285–295

### 4.2 Isotonic resuscitation fluids

The aim of resuscitation fluids in the context of anesthesiology is pivotal as they serve to replenish and optimize the ECF compartment (4). General anesthesia triggers a cascade of physiological changes, including suppression of the adrenergic nervous system. This suppression manifests as widespread vasodilation, leading to transient shifts in the fluid distribution throughout the body (19, 28). Specifically, these shifts entail the augmentation of the “unstressed” blood volume at the expense of the “stressed” blood volume. The former refers to blood that is not under immediate pressure against the venous walls, whereas the latter represents blood that is actively engaged in exerting pressure within the circulatory system (81). Additionally, this redistribution correlates with a decline in the mean circulatory filling pressure, which consequently diminishes the overall driving pressure for the circulation. Remarkably, anesthesia typically induces a reduction of approximately 25% in this pressure, translating to a relative deficit of approximately 1.2 liters of blood (82). Such a deficit instigates a cascade of interrelated hemodynamic adjustments. Venous return and cardiac output are reduced, thereby precipitating a decline in the arterial pressure (38). However, microcirculation is modestly enhanced by general anesthesia (83) as long as blunt shock does not result in compromised perfusion, leading to diminished blood supply to vital organs, including the GI tract and the kidneys (84). Likewise, the cerebral blood flow remains relatively preserved, particularly when the patient is maintained in a recumbent position (85).

Effective fluid therapy during surgery is paramount to counteract these intricate circulatory dynamics. However, compensating for the relative fluid deficit of 1.2 liters with only crystalloid solutions poses a significant challenge. Furthermore, excessive use of colloids may increase the risk of pulmonary edema during the postoperative phase, given the transient nature of augmentation of “unstressed” blood volume. Moreover, the diuretic response to fluid administration, which is significantly blunted during surgery, typically normalizes soon after emergence from anesthesia, facilitating hemodynamic recovery.

The anesthetist’s responsibility lies in strategically mitigating the effects of attenuated adrenergic tone through judicious fluid management, possibly augmented by the administration of vasoconstrictors when deemed necessary. In addition to addressing the relative deficit incurred during induction, supplementary fluid is indispensable to offset the ongoing surgical blood loss and mitigate the evaporative losses encountered during the procedure (86).

#### 4.2.1 Isotonic (0.9%) sodium chloride

0.9% sodium chloride is an isotonic solution that is renowned as a cornerstone crystalloid solution in clinical practice. It is widely utilized for volume replacement across various medical scenarios and is the preferred fluid choice in many clinical contexts. However, it is imperative to recognize that the terms “normal” or “physiological” saline are misleading. While its osmolarity hovers around 300 mOsm/L, it is crucial to understand that this designation is derived from its concentration of sodium chloride, not necessarily because its composition mirrors physiological electrolyte levels. This solution, crafted by rendering a sterile water solution isotonic through the addition of sodium chloride, presents a notable discrepancy in chloride concentration compared to bodily fluids. Infusion of 2 liters or more of 0.9% sodium chloride leads to hyperchloremic metabolic acidosis, primarily due to the elevated chloride content relative to the body’s normal physiological levels (87, 88).

Despite this drawback, 0.9% sodium chloride is indicated in scenarios, such as hypochloremic metabolic alkalosis, which is commonly observed in cases of vomiting. Furthermore, its sodium content, which is slightly higher than that of plasma, makes 0.9% sodium chloride beneficial for correcting hyponatremia (15).

Recent population studies, based on large numbers of patients, failed to associate 0.9% sodium chloride with more major complications than balanced fluids when used in daily routine (73, 89–91), the only possible exception being in septic patients (92). However, it is essential to acknowledge the limitations of 0.9% sodium chloride. Although it remains a staple resuscitation fluid during surgery, even in instances where balanced solutions might be pharmacologically superior, its use has been associated with certain drawbacks. Research indicates that electrolyte-based crystalloid fluids, such as balanced solutions, show promise in mitigating the risks associated with the use of 0.9% sodium chloride, including renal dysfunction, heart failure, and when there is risk of cardiac arrest (93). Nevertheless, further investigation is warranted to comprehensively address these limitations and to provide stronger evidence to support their efficacy.

#### 4.2.2 Ringer solutions

Ringer’s solution, pioneered by Sydney Ringer in the 1880s, was crafted with the intention of closely resembling the composition of ECF. Later modifications by Hartmann, who introduced the buffering agent, lactate, resulted in a formulation known as “Hartmann’s solution” or “Lactated Ringer’s solution.” Ringer’s solution may also be used with acetate as buffer. Both ions are metabolized in the body and ultimately converted to bicarbonate. While the differences between lactate and acetate are typically minimal, acetate is considered the preferred buffer in cases of compromised circulation, such as shock.

Ringer’s solution is slightly hypotonic. Complete distribution from the plasma to the interstitial fluid space occurs over a period of 25–30 min (17). Elimination is prompt in awake subjects (half-life 30–50 min) but occurs much more slowly during general anesthesia (−85 to −90%) (24). Transcapillary leakage of Ringer accelerates lymphatic return within minutes because fluid shifts in the fast-exchange interstitial space operate like a telephone line, although individual molecules travel much more slowly. A delicate balance between capillary filtration and lymphatic flow soon stabilizes plasma volume expansion at a steady-state level, typically corresponding to 20% of the infused volume. However, large amounts of fluid open an additional and more remote secondary interstitial space (“third space,” *V*_t2_) which seems to be a mechanism that prevents circulatory overload (18).

#### 4.2.3 Plasma Lyte

Plasma-Lyte® represents a contemporary addition to the family of electrolyte-based crystalloid fluids, boasting an enhanced electrolyte composition that aligns even more closely with the electrolyte concentrations found in ECF. To achieve this precise balance, Plasma-Lyte® incorporates both acetate and gluconate. Gluconate, often employed as an inert ion in the food industry for its taste-enhancing properties, plays a pivotal role in harmonizing the chloride concentration within Plasma-Lyte® with that of plasma, thereby mitigating the risk of metabolic acidosis. Benefits include that Plasma-Lyte® is iso-osmotic, the slightly higher sodium concentration (140 mmol/L) compared to Ringer’s makes post-infusion hyponatremia unlikely. The fluid lacks calcium, which allows it to be used together with erythrocyte concentrates using citrate as the anticoagulant without risk of coagulation in the infusion line. Large amounts of Plasma-Lyte® (4 L), like 0.9% sodium chloride, dilutes plasma calcium enough to weaken cardiac pumping, but this is easily reversed by giving an intravenous injection of calcium. However, Plasma-Lyte® has a mild alkalinizing effect in clinical practice, due to its strong *in vivo* ion difference (SIDe) of 50. Consequently, cautious administration is warranted in alkalotic patients, particularly when considering large-volume infusions, as this could exacerbate the alkalinizing effects by dilution of weak acids (94).

Balanced solutions containing acetate have garnered significant attention. Observational data suggest that these solutions maintain acid–base balance more effectively than 0.9% sodium chloride, particularly in the context of major abdominal surgery and transplantation. However, investigations of the compatibility of Plasma-Lyte^®^ with various drugs have revealed certain interactions, including increased turbidity observed when mixed with propofol, amiodarone, cyclosporine, and mycophenolate. Conversely, it demonstrated stability with furosemide at lower concentrations than those typically used in standard perfusion practices. Notably, Plasma-Lyte^®^ can enhance the excretion of acidic drugs, such as barbiturates, while concurrently diminishing the renal excretion of sympathomimetics (e.g., ephedrine) due to its alkalinizing effects (95).

Despite its versatility, Plasma-Lyte^®^ is not without limitations. Its use in the ICU may yield false-positive results for galactomannan antigen, a biomarker of aspergillosis. However, modern units have devised sophisticated confirmation procedures to effectively circumvent this issue. Approved by the Food and Drug Administration (FDA) for use in hemodialysis, Moreover, it is recommended as a balanced solution for perioperative fluid therapy in patients with diabetes, particularly in those with poor glycemic control, and in individuals undergoing major liver surgery.

#### 4.2.4 Isofundin/Sterofundin/Ringerfundin

Isofundin^®^ has emerged as a noteworthy addition to the group of balanced crystalloid fluids, boasting a buffering system that incorporates both acetate and malate. Notably, the calcium and magnesium contents closely mirror those of plasma, underscoring its potential to maintain electrolyte balance. However, the sodium and chloride concentrations surpass those found in plasma, offering a hyperosmolar solution that is distinct from its counterparts. Depending on the European country, this fluid is marketed under different names, such as Isofundin^®^, Sterofundin^®^ or even Ringerfundin^®^.

Despite its calcium content, akin to that of Ringer’s solution, Isofundin® poses compatibility challenges with citrate-preserved blood products. The limited literature available on this crystalloid underscore its relatively understudied nature, although it has featured comparative studies alongside other balanced solutions. Notably, Plasma-Lyte^®^ and lactated Ringer’s solutions have a lower chloride content, rendering them more balanced alternatives compared to Isofundin^®^ (94, 95). One study comparing Isofundin^®^ with Plasmalyte® observed a decline in serum urea levels following Isofundin^®^ administration, which was attributed to enhanced renal excretion and sustained calcium levels. In contrast, Plasmalyte® was associated with a trend toward decreased serum calcium levels following 2 liters administration in healthy volunteers (96). In a comparative study examining Sterofundin^®^ and lactated Ringer’s solution in scoliosis surgery, participants administered Sterofundin^®^ exhibited higher plasma chloride concentrations. However, stability in pH and base excess was maintained, which was attributed to the elevated strong ion difference that compensates for hyperchloremia (97).

Other crystalloids with potential use as resuscitation fluids are shown in Table 2.

**Table 2:** Crystalloids with potential use as resuscitation fluids.

	NaCl 0.9 %	Lactated Ringer’s	Plasmalyte^®^	Isofundin^®^ Sterofundin^®^	Ionolyte^®^	Plasma
Na^+^ (mEq/L)	154	131	140	145	137	140
K^+^ (mEq/L)	–	5.4	5	4	4	4
Ca^2+^ (mEq/L)	–	2	0	2.5	0	5
Mg^2+^ (mEq/L)	–	–	1.5	1	1.5	2.5
Cl^-^ (mEq/L)	154	109	98	127	110	98
Bicarbonate (mEq/L)	–	–	–	–	–	24
Lactate (mEq/L)	–	28	–	–	–	–
Acetate (mEq/L)	–	–	27	24	34	–
Citrate (mEq/L)	–	–	–	–	–	–
Malate (mEq/L)	–	–	–	5	–	–
Gluconate (mEq/L)	–	–	23	–	–	–
Glucose (g/L)	–	–	–	–	–	–
SID *in vivo* (mEq/L)	0	28	50	25.5	34	42
Osmolarity	308	277	295	309	286.5	285–295
(mOsm/L)						

#### 4.2.5 Colloid fluids

The term *colloid fluid* implies that a macromolecule that passes through the capillary wall only with difficulty has been added to an electrolyte solution (usually 0.9% sodium chloride). The minor contribution of this macromolecule to the osmolality is sufficient to retain the infused fluid in the bloodstream. The macromolecule finally distributes to the interstitial space or becomes metabolized, whereby the plasma volume expansion subsides. However, the duration of the plasma volume expansion induced by a colloid fluid is much longer than that of a crystalloid fluid, at least for up to 6–8 h (cf. Figure 3 with Figure 4).

Colloid fluids have been used less frequently in recent years (98) as researchers have questioned their usefulness. Their current role is to increase plasma volume expansion effectively in hypovolemic patients. In particular, colloids may be used expand the plasma volume beyond what can be achievable with 3–4 L of crystalloid fluid, which is when crystalloid-associated adverse effects begin to develop (99). In this way, the total number of adverse effects of the fluid therapy can be limited (8). However, this is only achievable in patients with a normal or high blood hemoglobin concentration where administration of 3 L of crystalloid might place the patient close to his/her individual “transfusion trigger” which is when erythrocyte transfusion becomes indicated. The macromolecules in colloid fluids can induce allergic reactions, of which the anesthetist needs to be prepared. These are rare events but is one reason to why the use of colloids is limited.

The colloid fluids are either natural (5 and 20% human albumin) and synthetic (hydroxyethyl starch, gelatin, and dextran). Albumin is currently the most widely accepted colloid in the clinic. The hyper-oncotic preparation, albumin 20%, withdraws interstitial fluid to the plasma, which primarily occurs via the lymphatic system (100). Every milliliter of infused albumin 20% recruits 3.4 times the infused volume of which 2/3 is excreted as urine (101). Hence, the plasma volume is expanded by twice the infused volume (29, 30) Albumin 20% also has a diuretic effect that is only marginally impaired by hypotension, which is not the case for crystalloid fluid (102). Therefore, albumin 20% would be an effective treatment in hypovolemic, hypotensive patients with peripheral edema. Interestingly, the capillary leakage of the albumin molecules does not seem to be accelerated despite high plasma concentrations levels of shedding products from the endothelial glycocalyx layer (35).

Hydroxyethyl starch 130/0.4 (Voluven^®^) was long favored as a less expensive synthetic alternative to albumin but became the subject of restrictions a decade ago when an increased incidence of kidney failure was detected in septic patients. However, no increased incidence of renal problems has been found in patients undergoing elective surgery (103–109). This colloid is still widely used in Asia while its use in Europe and the US has been declining. Voluven still has a role as infusion fluid when performing “goal-directed fluid therapy” (see “Fluid strategies during surgery” below). The early studies evaluating this approach (1995-2012) showed that it reduces number of postoperative complications, which is probably due to a consistent and long-standing plasma volume expanding effect. Later studies, performed after hydroxyethyl starch fell out of favor, have shown mixed results, but are based on using Ringer solutions which have a different pharmacokinetic profile that involves “attenuation” upon repeated administration (110).

#### 4.2.6 Hemodynamic monitoring

Hemodynamic monitoring during surgery has conventionally consisted of non-invasive arterial pressure, heart rate, and urine output. These parameters are influenced by variables unrelated to circulatory status, making them unreliable for assessing intravascular volume. Furthermore, they do not provide information on the balance between oxygen supply and demand. Their utility is limited to specific clinical scenarios, as they only offer data about the cardiovascular system under certain conditions (111). Because of these limitations, in recent decades, many monitoring tools has become available for the anesthetic team, allowing treatment to be tailored to the patient’s physiology, either with fluids, vasoactive agents or even ionotropic agents (112).

Hemodynamic monitoring devices can be classified in many ways; one of the most common is by degree of invasiveness. According to this criterion, there are **invasive methods** (*pulmonary artery catheter and transpulmonary thermodilution*), **minimally invasive systems** (*lithium dilution, pulse wave contour analysis*) and **non-invasive** (*ultrasound, bioimpedance, among others*) (113).

##### 4.2.6.1 Invasive and minimally invasive methods

Transcardiac thermodilution using a pulmonary artery catheter (PAC) has been considered the reference method for measuring cardiac output (CO) since its introduction in the 1970s. Although most CO estimation techniques have been validated against the PAC, it is not without limitations. The PAC enables the measurement of important hemodynamic parameters, such as pulmonary artery occlusion pressure (PAOP); however, its invasive nature and ongoing debates regarding its indications and potential complications have led to a decline in its use over recent decades (38).

Transpulmonary thermodilution is a less invasive alternative. In this method, a cold fluid bolus is injected through a central venous catheter, and a sensor placed in the femoral artery detects the temperature change (external calibration). Cardiac output is then calculated using the Stewart-Hamilton equation. The accuracy of this technique may be affected by fluctuations in body temperature or the presence of intracardiac shunts (114).

Another externally calibrated method is the LiDCO system, which uses a lithium bolus injected into the circulation. The time-concentration curve is measured in a peripheral artery, after which a pulse-contour analysis provides continuous CO monitoring (115).

Pulse-contour analysis (PCA) estimates cardiac output and other hemodynamic parameters by analyzing the shape of the arterial pulse waveform. Its accuracy improves when calibrated with thermodilution, as in systems like PiCCO (Pulsion) and VolumeView (Edwards). Some minimally invasive devices, such as Vigileo/FloTrac (Edwards) and MostCare (Vygon), do not require external calibration, although this may slightly reduce measurement accuracy (116).

Additionally, arterial waveform analysis based on heart-lung interactions provides dynamic variables that help predict fluid responsiveness, such as stroke volume variation (SVV) and pulse pressure variation (PPV). These variables do not require fluid challenges but are only applicable in mechanically ventilated patients. They are incorporated into many commercially available hemodynamic monitoring systems (117).

##### 4.2.6.2 Non-invasive monitoring

There are methods that guide fluid therapy without needing access to the vascular system. Esophagus-Doppler (Deltex) uses a probe placed in the esophagus where CO is derived from the flow in the descending aorta (118). Another example is ClearSight (Edwards), which estimates central circulation variables based on recording of the pulse wave in a finger (119). Pleth variability index (PVI) is a non-invasive variant of PPV implemented on Masimo pulse oximeters (120).

The state of the central circulation can also be assessed by thoracic bioimpedance as, for example, with NICOM (Cheetah). Breathing-induced variations of the width of the inferior cava vein can be quantified by ultrasound, which provides information about vascular filling and fluid responsiveness (121).

A simple way to assess fluid responsiveness is to raise the legs, which redistributes blood from the legs to the central circulation by gravitational forces (“passive leg raising test”). This creates a temporary increase of venous return which is maintained as a long as the legs are raised. However, the stroke volume response must still be assessed using one of the methods mentioned above. This test is difficult to perform in the operating room and is better suited for pre- and postoperative care (122).

Lung maneuvers have also been explored as methods to assess fluid responsiveness. However, a detailed description of them and the different approaches to challenge fluid responsiveness is beyond the scope of this review. The interested reader is referred to a review (123).

### 4.3 Replacement fluids

Replacement fluids play a pivotal role in restoring fluid deficits that cannot be remedied through oral intake, which may arise from a myriad of conditions, such as vomiting, diarrhea, or other GI losses (124). These deficits can lead to dehydration, electrolyte imbalance, and compromised hemodynamic stability, underscoring the need for prompt and tailored fluid replacement strategies (125).

The selection of the appropriate replacement fluid depends on an understanding of the underlying etiology of the fluid deficit. For instance, isotonic balanced solutions are typically preferred in cases of isotonic fluid loss, such as ECF depletion due to vomiting or diarrhea. These solutions mimic the electrolyte composition of the extracellular fluid, facilitating rapid restoration of fluid balance without causing significant shifts in osmolarity or electrolyte concentrations. In contrast, patients with deficits stemming from specific electrolyte losses may benefit from targeted replacement strategies. For example, patients experiencing chloride-rich fluid loss, such as gastric fluid loss from nasogastric suctioning or prolonged vomiting, may require high-chloride solutions such as 0.9% sodium chloride for effective replacement. These solutions not only replenish the fluid volume but also help restore the electrolyte balance by addressing specific ion losses (62). Moreover, the volume and rate of fluid replacement must be carefully titrated according to the patient’s clinical status, volume deficit, and ongoing losses. Close monitoring of vital signs, electrolyte levels, and hemodynamic parameters is essential to guide the fluid management to prevent complications, such as fluid overload or electrolyte disturbances (126).

Overall, a comprehensive understanding of the underlying pathophysiology of fluid deficits coupled with judicious selection and administration of replacement fluids is necessary to optimize patient outcomes and restore hemodynamic stability in cases of dehydration or electrolyte imbalances (127).

### 4.4 Nutrition fluids

Nutritional therapy, primarily administered via the enteral route, alleviates stress-induced metabolic responses, prevents oxidative cell damage, and positively modulates immune reactions. Recently, guidelines have replaced the term “*artificial nutrition*” with “*medical nutrition therapy*” encompassing oral nutritional supplements, enteral nutrition, and parenteral nutrition (128).

The ASPEN guidelines recommend considering supplemental parenteral nutrition if >60% of the energy and protein needs cannot be met by enteral nutrition alone after 7–10 days in patients with low or high nutritional risk. Early initiation of supplemental parenteral nutrition in critically ill patients receiving some enteral nutrition does not improve the outcomes and may be harmful. For low-risk patients unable to maintain intake or initiate early enteral nutrition, exclusive parenteral nutrition should be avoided in the first 7 days following ICU admission (129).

In high-risk or severely malnourished patients necessitating parenteral nutrition, hypocaloric dosing (≤20 kcal/kg/day or 80% of the estimated energy need) with adequate protein (≥1.2 g protein/kg/day) is recommended initially over the first week in the ICU. Postoperative enteral nutrition within 24 h of surgery is preferred over parenteral nutrition. Immune-modulating formulas, particularly those containing arginine and fish oils, are recommended postoperatively in patients requiring enteral nutrition (129).

In cases where enteral nutrition is infeasible after major upper GI surgery, early initiation of parenteral nutrition is advised if the therapy duration exceeds 7 days. Careful fluid management is crucial during parenteral/enteral nutrition considering the hyperosmolar nature of parenteral nutrition and its potential complications (electrolyte disturbances and hyperglycemia) (130).

Regular electrolyte checks are necessary, particularly in patients receiving enteral or parenteral nutrition. Standard total parenteral nutrition solutions typically contain sodium (30–80 mEq/L), potassium (30–40 mEq/L), magnesium (4–12 mEq/L), and phosphate (10–15 mmol/L), necessitating replacement with parenteral therapy (131).

Parenteral nutrition, administered intravenously to individuals who are unable to tolerate oral or enteral feeding, provides comprehensive nutrient support tailored to individual needs. Careful monitoring and adjustment of nutritional support are crucial to mitigate fluid imbalances and optimize patient outcomes given the potential exacerbation of fluid overload or accumulation with parenteral nutrition (129).

## 5 Fluid strategies during surgery

The relationship between intraoperative fluid volume and postoperative complications follows a nuanced pattern, resembling a “U-form.” This indicates that complications become more prevalent when too little fluid or too much fluid is administered. However, determining the optimal fluid volume is a multifaceted challenge as individual patient needs vary greatly (1). Consequently, intraoperative fluid therapy has emerged as a complex intervention that operates at multiple levels that necessitates consideration of physiological principles and clinical context (132). The challenge is to use infusion fluids as a tool in the complex task of maintaining hemodynamic stability and good performance of the cardiovascular system, which includes the prevention of excessive vasoconstriction (“protective hemodynamics”). Fluid therapy is an integrated part of this work but is not an independent entity. Nevertheless, several approaches have developed which the clinician may consider when planning the fluid therapy during surgery.

One of the prevailing methods is the *fluid balance approach* wherein the anesthetist meticulously tracks the measured and estimated fluid losses during surgery and replaces these volumes based on the known plasma volume expansion effects of crystalloid and colloid infusion fluids. This personalized method is commonly employed, particularly in surgeries of short durations (133).

The *outcome-based approach* means relying a fluid program based on published studies that compare postoperative complication rates between groups of patients randomized to receive different predetermined fluid regimens, such as liberal/restrictive or crystalloid/colloid strategies (134). This approach may be suitable for intermediate-length surgeries (1–3 h) without significant hemorrhage, and over the past two decades, numerous studies have compared various intraoperative infusion protocols using multiple definitions of liberal and restrictive regimens. Typically, a regimen in which fluid is administered faster than 7 mL/kg/h during abdominal surgery is categorized as being liberal, whereas infusion rates not surpassing 5 mL/kg/h are considered restrictive (7).

Early outcome-oriented studies focused on abdominal surgery and showed that a fluid administration rate of 3-5 mL/kg/h was associated with more favorable outcomes than twice as high rate (37). Complications usually occur where crystalloid fluid is known to end up, which is primarily in the skin, GI tract, and lungs [6]. A compilation of outcome studies identified 2.8 L as the optimal fluid volume during abdominal surgery (135); hence, complications were more common when less or more volume was administered. The first sign of crystalloid fluid overload is the long-standing inhibition of GI movements (paralytic ileus), which becomes common when more than 2 L of fluid is infused (136). One of the few studies that found a benefit of large fluid volumes compared to low volumes (6 L vs. 3 L) is the RELIEF study, in which the authors formulated an outcome-oriented recommendation for major surgery (10). However, for major surgeries, most researchers recommend individualized fluid therapy guided by hemodynamic measurements, and not the use of an outcome-based approach (137).

*Goal-directed fluid therapy (GDFT)* represents a tailored approach that is particularly pertinent to major surgeries with anticipated substantial hemorrhage. Although colloid fluids have historically been favored for this purpose, contemporary practice leans toward crystalloid fluid administration. GDFT standardizes hemodynamic goals through therapeutic protocols, typically aiming to optimize flow-related parameters, such as cardiac output (CO) or stroke volume, with infusion fluid boluses, possibly supplemented with vasopressors (138). GDFT assumes that optimal vascular volume improves cardiac pumping, being “optimal” when enough volume is provided to reach the flat part of the Frank-Starling curve. This optimization of the relationship between blood volume and CO should be reflected in improved microcirculation without the oxygen diffusion to tissues being reduced (139).

Studies from the 1990s showed clear benefits of GDFT regarding postoperative complications and survival, while the experience from the past decade has been mixed. One possible reason for this change is that control programs have improved over time, as they often incorporate experiences from outcome-oriented studies. Another possibility is that the changeover over from colloid to crystalloid fluid for the fluid challenges, which occurred a decade ago, reduced the efficacy of GDFT due to the pharmacokinetic differences between the two fluids (110).

Systematic reviews have usually found that GDFT reduces the length of hospital stay and the overall rate of postoperative complications. Estimates of mortality and organ-specific complications tended to favor GDFT with variable degrees of accuracy (38). For example, a recent meta-analysis including 76 studies showed that GDFT might reduce mortality (odds ratio = 0.84; 95% CI: 0.64–1.09) and shorten the hospital stay (mean difference = −0.72 days; 95% CI: −1.10 to −0.35), but with low certainty in the evidence (99). A problem when conducting meta-analyses of this topic is the large number of protocols and hemodynamic monitors in use, which complicates comparisons. Moreover, protocols may differ in terms of how complications of moderate severity are recorded.

Although the GDFT concept is primarily based on flow optimization, more recent hemodynamic algorithms add a target for the mean arterial pressure (MAP) of ≥65 mmHg or individually determined from preoperative baseline MAP to be achieved using vasopressors (140). In general terms, the strategy is to optimize flow by fluid administration and then to correct hypotension, if present.

Some hemodynamic algorithms include target cardiac index (CI) values, which imply the administration of inotropes in non-preload-dependent patients. In contrast to the “one-size-fits-all” approach, a personalized CI strategy could be more beneficial (141). In high-risk patients undergoing major abdominal surgery, personalized hemodynamic management comprising strategies to maintain baseline CI using a GDFT algorithm decreased the composite outcome of major postoperative complications or death within 30 days after surgery compared to usual care (relative risk: 0.54, 95% CI: 0.38–0.77; absolute risk reduction: −25.5, 95% CI: −39.2 to −11.9%; *p* < 0.001) (142).

The selection of hemodynamic targets remains controversial, but there is a consensus that both adequate arterial pressure and blood flow are essential determinants for maintaining the microcirculation; that is, acceptable MAP and CO are both required to meet the body’s total oxygen requirements. Therefore, monitoring MAP and systemic blood flow on a beat-by-beat basis would ideally help to optimize tissue perfusion (143).

Conclusions about the clinical value of GDFT are hampered by the variability in the protocols used and the frequently poor agreement between methods used to assess the hemodynamic response (“fluid responsiveness”). GDFT is time-consuming and requires invasive hemodynamic monitoring and seems best suited for major surgery in high-risk patients.

In general, the indication for invasive hemodynamic monitoring becomes stronger with more extensive surgery and poorer health status of the patient. Here, the margin for non-optimal management is slim and the risk of iatrogenic complications greater.

## 6 Pediatric perioperative fluid therapy

Pediatric fluid therapy aims to provide the necessary fluid volume to maintain tissue perfusion, meet daily fluid, glucose and electrolyte requirements, correct any existing deficits, and nourish the patient when oral intake is not possible (144, 145).

Pediatric patients differ from adults: there are anatomical and physiological changes that occur throughout childhood and early years of life due to growth and organ maturation (146). Therefore, the same guidelines as those for adults cannot be applied, and fluid therapy must be tailored to various stages of pediatric age because dosage errors or inappropriate use of fluid therapy can lead to life-threatening complications. Surgical scenarios and critical events often lead to fluid loss owing to bleeding, loss of surgical viscera exposure related to mechanical ventilation, and increased metabolism. Conversely, excessive fluid infusion can occur if the maintenance infusion is not adjusted according to the patient’s age and weight or if the additional volume contributed by drug infusions (fluid creep) is not considered.

There are five aspects to be considered when planning a perioperative fluid therapy in pediatric patients. First, the patient’s volemic status, which may be affected by prolonged fasting, must be known. Second, the addition of glucose is not always necessary, and it must be tailored to the patient specific needs. Third and fourth, it is important to select the appropriate tonicity of the fluid, recommending the use of balanced buffered solutions. Finally, the rate of infusion must be also tailored to both weight and type of surgery because both hypovolemia and hypervolemia are associated with perioperative complications, as it is in adults.

### 6.1 Preoperative fasting and fluid deficits

Pediatric patients poorly tolerate prolonged fasting owing to the risk of dehydration and hypoglycemia, resulting in metabolic and neurohormonal changes that may lead to ketosis and catabolism. Prolonged fasting increases the risk of hypotension during anesthesia induction (147). Hypovolemia is the most common cause of perioperative cardiac arrest in children (148). Additionally, extended fasting periods can cause anxiety and irritability in both the children and their caregivers. Promoting fluid intake and avoiding prolonged fasting can reduce postoperative nausea and vomiting and maintain hemodynamic stability (149).

Minimizing fasting and re-establishing early oral and enteral intake are key objectives in perioperative and critical care settings. The risk of aspiration in the pediatric population is low (1-10:10,000), with extremely low morbidity and mortality rates (150). Gastric emptying of fluids follows an exponential curve, unlike solids, which maintain a steady pace, resulting in rapid gastric emptying, with 5 mL/kg of clear fluid eliminated within an hour. The European Society of Pediatric Anesthesia recommends ≤ 3 mL/kg of clear fluid based on magnetic resonance findings (151).

The European Society of Anesthesiology and Intensive Care (ESAIC), Association of Pediatric Anaesthetists of Great Britain and Ireland, Australian and New Zealand College of Anaesthetists, European Society for Pediatric Anesthesiology, and Association des Anesthésistes Réanimateurs Pédiatriques de l’expression Française recommend fasting times of 1 h for clear oral fluids (including glucose-containing isotonic drinks) before elective procedures in children (151). However, the 2023 American Society of Anesthesiologists practice guidelines for preoperative fasting do not provide sufficient evidence regarding the benefits and harms of recommending 1 h versus 2 h of fasting (152).

### 6.2 Maintenance fluid therapy

The ionic compositions of the plasma and extracellular fluid are similar across all ages, allowing the use of fluids with the same electrolyte composition as that used in adults. Balanced isotonic crystalloids are first-line fluids for intraoperative maintenance and volume replacement in the pediatric population (150).

#### 6.2.1 Glucose solutions

The addition of glucose remains a key factor in pediatric fluid therapy. Both hyperglycemia and hypoglycemia are associated with morbidity and neurological damage in the pediatric population; the goal is to achieve a glucose concentration of 5–10 mmol/L (153).

During anesthetic procedures, there is a decrease in metabolism and insulin resistance secondary to surgical stress, which reduces the usual glucose requirements of hospitalized children. However, insufficient glucose supplementation (for example, long preoperative fasting times or in at-risk patients) increases the risk of lipolysis with the release of free fatty acids and ketone bodies, leading to hypoglycemia, reduced base excess, and even ketoacidosis (147). Nevertheless, after the neonatal period, healthy children usually have sufficient metabolic reserves to maintain normoglycemia during most surgeries, with the risk of preoperative hypoglycemia ranging from 0 to 2.5%, which is related to prolonged fasting (147, 154).

To avoid altered glycemic states, recent guidelines recommend adding glucose at 1-2.5%, which is usually sufficient to prevent ketosis in young and preschool children up to 5 years of age (153). Balanced crystalloids with 1% glucose are available in the market, such as Benelyte^®^ (Fresenius), which combines 1% glucose in a balanced solution with Na^+^, K^+^, Cl^−^, and Mg^++^. Alternatively, they can be prepared by adding glucose to balanced solutions, although this is not recommended in terms of safety because dosing errors may occur.

The 1–2% glucose supplement prevents hypoglycemia but does not meet nutritional needs. Balanced isotonic solutions with glucose between 2 and 4% are more appropriate than 1% for the youngest age group undergoing major pediatric surgeries to avoid catabolism, insulin resistance, rebound hyperglycemia, and acidosis (153). The addition of glucose up to 5–10% is usually reserved for patients with low glycogen reserves, such as premature and neonatal infants, malnourished patients, situations of low CO, liver disease, states with high catabolic states, mitochondrial disorders, those receiving beta-blocker therapy, and those who are removed from parenteral nutrition during surgery (154).

#### 6.2.2 Hypotonic versus isotonic solutions

Holliday and Segar described the fluid and energy requirements of healthy children using a formula based on the daily caloric needs per kilogram and requirements for sodium, potassium, and chloride (155). Until a few years ago, this formula was used to prescribe fluid therapy for hospitalized children, and hypotonic crystalloids containing glucose, such as 0.2% NaCl or 5% dextrose water, have been the fluids of choice (155). This practice was also based on the conceptual understanding that maintenance fluids must hydrate both the extracellular and intracellular compartments, justifying the use of hypotonic solutions (sodium content < 70 mEq/L), along with the belief that children’s immature kidneys cannot excrete sodium (147). However, an alarm was raised in 1992 by reports of children with cerebral edema and deaths secondary to iatrogenic hyponatremia related to the administration of hypotonic fluid. This has led to a paradigm shift toward the use of isotonic fluids in the pediatric perioperative population (156).

Iatrogenic hyponatremia is relatively frequent in the pediatric perioperative population because of its association with several factors, such as increased secretion of vasopressin (antidiuretic hormone, ADH) in sick children due to non-osmotic stimuli (pain, stress, nausea, anxiety, and opioids) and/or intraoperative hypovolemia, which, together with the infusion of hypotonic fluids during the perioperative period, leads to excess free water (157). These mechanisms account for the current recommendation to place more emphasis on isotonic rather than hypotonic fluids during the perioperative period. The volume of hypotonic fluid given perioperatively was probably excessive in many cases, as there were periods in which the fluid infusion was not registered or was uncontrolled; for example, when moving to another unit without considering previous fluid infusion (operating room to postoperative care, and postoperative care to wards) or flushing the iv lines after administering medication to check its permeability. Additionally, there is increased neurological vulnerability in prepubescent children owing to their brain-to-bone size ratio, reduced Na-K-ATPase activity, and higher levels of the vasopressin in response to stress. Stress in these patients also activates the hypothalamic–pituitary–adrenal axis, stimulating aldosterone secretion which retains sodium and water (158). Hyponatremia might cause encephalopathy, leading to cerebral edema and intracranial hypertension, which can ultimately result in cardiorespiratory arrest and death. Early symptoms are nonspecific and frequent postoperatively, such as headache, nausea, vomiting, and lethargy, making early diagnosis challenging, but important, as early correct treatment ensures the best outcome (159).

A recent meta-analysis compared the risk of hyponatremia and hypernatremia in hospitalized children receiving both fluid therapies and found that isotonic fluids significantly decreased the risk of moderate hyponatremia, although they also found an increased risk of hypernatremia in neonates (158). The American Academy of Pediatrics and NICE recommended the use of isotonic solutions for intravenous maintenance fluid therapy in 2018 and again in 2023 in hospitalized pediatric patients > 1 month of age, except in cases with free water loss (160).

Pediatric patients are more susceptible than adults to developing symptoms, particularly neurological symptoms, due to hyponatremia. Nevertheless, we must carefully evaluate whether isotonic maintenance solutions are recommended for all hospitalized patients to prevent hyponatremia in a small subset of patients. This is especially questionable in the ICU setting, where other sodium sources are present, and sodium is measured multiple times daily (161). Moreover, there is limited evidence of clinically significant hyponatremia caused by maintenance fluid administration. In a key pediatric study on this topic, no patient developed symptomatic hyponatremia (162). Seizures occurred in seven out of 338 patients (2%) in the hypotonic group compared to one out of 338 patients (0.3%) in the isotonic group (*p* = 0.07), but all cases were in patients with pre-existing seizure disorders.

#### 6.2.3 Balanced solutions vs. 0.9% sodium chloride

The use of balanced isotonic crystalloids with ionic content similar to that of plasma and organic anions as bicarbonate precursors is recommended (157). The European consensus statement in 2011 and the guidelines from the Association of the Scientific Medical Societies in Germany in 2016 also recommend the use of isotonic balanced crystalloids containing glucose at 1–2.5% for maintenance fluid therapy in children up to 5 years of age (156). Amer et al. suggested the use of balanced solutions over those with a high chloride content, such as 0.9% sodium chloride, to avoid the risk of renal dysfunction associated with intravenous fluid therapy, especially in neonates (158).

The addition of potassium to maintenance fluids is advisable. A content of 4–5 mmol/L is usually sufficient for most patients; however, certain clinical conditions, such as acute renal failure, crush syndrome, and tumor lysis syndrome, may present with hyperkalemia, and it is advisable to minimize or avoid potassium content in maintenance fluids. Conversely, there are clinical circumstances in which potassium depletion should be suspected and appropriately replaced upon confirmation, including diabetic ketoacidosis, diuretic use, and insulin therapy (163).

Addition of calcium and magnesium is necessary in patients with prolonged maintenance fluid therapy, such as those in the ICU, and requires laboratory monitoring.

#### 6.2.4 Volume and rate of infusion

The National Institute for Health and Care Excellence (NICE) guidelines recommend using the Holliday and Segar equations to calculate basal daily volume fluid requirements. This is known as the “4-2-1 rule,” as outlined in Table 3.

**Table 3:** Basal fluid requirements calculation 4-2-1 by Holliday-Segar.

Weight	Fluids per hour
0–10 kg	4 mL/kg/h
10–20 kg	40 mL/h + 2 mL/kg/h for each additional kg > 10 kg
>20 kg	60 mL/h + 1 mL/kg/h for each additional kg > 20 kg

The “4-2-1 rule” is used to calculate the hourly fluid maintenance rate in children: 4 mL/kg/h for the first 10 kg and additional 2 mL/kg/h for the next 10 kg, and finally 1 mL/kg/h for each kg above 20. This formula was originally designed to calculate the requirements of healthy children outside the perioperative setting. However, in surgical environments and ICUs, losses may escalate owing to visceral exposure, hemorrhage, edema, increased consumption, and mechanical ventilation, necessitating adjustments in fluid and electrolyte provision to suit the clinical context. Adjustments may include calculating losses ranging from to 1-2 mL/kg/h in superficial surgeries and escalating to 7–10 mL/kg/h in major surgeries involving extensive visceral exposure (156). Other authors have suggested a replacement rate based on the “4-2-1” rule supplemented with an additional rate of 2-4-6 mL/kg/h of balanced solution depending on whether the surgery is minor, intermediate, or major (147).

In cases of significant losses, these should be closely monitored and replaced with glucose-free balanced solutions or blood if necessary. This replacement should be accompanied by appropriate hemodynamic and gasometric monitoring to optimize the CO based on specific pediatric parameters (164). Utilizing flow rate control systems, such as volumetric infusion pumps or manual flow control systems is advisable. Infusion pumps should incorporate pressure alarms when used for neonates or infants. Infusion systems should always be checked for air bubbles to prevent air embolism.

Early postoperative oral tolerance is promoted to restore oral fluid needs, improve comfort, and facilitate early discharge in ambulatory surgery. For surgeries lasting less than 1 h who have consumed sufficient fluids before surgery, German guidelines from 2017, revised and agreed upon in 2021, suggest that fluid therapy is unnecessary even if intravenous access is required. However, fluid therapy should be administered if the patient is dehydrated (e.g., with prolonged fasting), regardless of the duration of the surgery, as well as in neonates, owing to their high caloric expenditure (154). The hydration status should be carefully assessed, as short periods of fasting with liquids often do not restore euvolemia because the volumes typically ingested are small and do not guarantee complete rehydration (160).

#### 6.2.5 Postoperative fluid management

During the postoperative period, early oral tolerance and enteral hydration should be encouraged as soon as possible, although caution should be exercised to avoid exacerbating vomiting (147). Until the oral intake is sufficient, fluid therapy should be maintained, focusing on both fluid loss and free water retention. The latter is favored in situations such as intravascular volume depletion, which is the most potent stimulus for release of vasopressin (antidiuretic hormone), or in situations of high perioperative stress such as major surgery associated with elevated vasopressin secretion. Therefore, reducing the rate of postoperative maintenance fluid therapy by 50-30% is recommended to prevent the risk of hyponatremia, or even up to 80% as recommended by the NICE guidelines, returning to the “4-2-1” rate once diuresis has normalized and there is no risk of elevated vasopressin (160). Additionally, the volume contributions associated with drug administration (fluid creep) and abnormal losses of organic fluids in cases of diarrhea, vomiting, biliary losses via the nasogastric tube, ileostomies, etc. should be considered (Table 4). These losses should be replenished at a rate of 1 mL of loss/1 mL of fluid, along with ionic losses associated with the type of organic fluid that originates them (potassium in the case of diarrhea).

**Table 4:** Composition of body fluids.

Fluid	Na^+^ (mEq/L)	K^+^ (mEq/L)	Cl^−^ (mEq/L)	HCO_3_^−^ (mEq/L)
Gastric	50	10–15	150	0
Pancreas	140	5	0–100	100
Bile	130	5	100	40
Ileostomy	130	15–20	120	25–30
Diarrhea	50	35	40	50
Sweat	50	5	55	0
Burnt skin	140	5	110	
Urine	0–100	20–100	70–100	0

Electrolyte status and acid–base balance should be monitored in all patients undergoing prolonged maintenance fluid therapy. In neonatal, premature, or metabolically impaired children, prolonged surgeries, hemorrhage, sepsis, significant losses, or other conditions, fluid intake and output should be monitored, daily weight should be recorded, and serial blood gas analyses should be performed to monitor the correct fluid and electrolyte balance and acid–base status of the patient (165).

### 6.3 Resuscitation fluid therapy

The 2023 update of the *Surviving Sepsis Campaign* (SSC) reflects a shift in approach by suggesting—rather than strongly recommending—the administration of at least 30 mL/kg of intravenous crystalloids within the first 3 h for adults with sepsis, emphasizing instead a more individualized strategy. This emerging approach, termed “glycocalyx resuscitation,” prioritizes treatment tailored to each patient’s fluid tolerance and responsiveness. In the pediatric population, the 2020 *SSC International Guidelines for the Management of Septic Shock and Sepsis-Associated Organ Dysfunction in Children* recommend the administration of up to 40–60 mL/kg of fluids during initial resuscitation, delivered in 10–20 mL/kg boluses over the first hour. Fluid administration should be guided by dynamic clinical markers of cardiac output and discontinued upon detection of fluid overload (166).

Recognizing clinical indicators of poor fluid tolerance is essential to avoid iatrogenic harm. Such signs include hepatomegaly, rales or crackles upon lung auscultation, increased work of breathing, generalized or facial edema, a sudden rise in heart rate, and lack of improvement—or worsening—of perfusion despite fluid therapy (167). These findings suggest a transition from fluid responsiveness to fluid intolerance, underscoring the importance of timely cessation of volume expansion to prevent adverse outcomes (168).

The 2021 *European Resuscitation Council (ERC)* pediatric guidelines introduced a more conservative approach, reducing the recommended fluid bolus from 20 mL/kg to 10 mL/kg. This change is aimed at minimizing risks associated with fluid overload, such as respiratory compromise and dilutional coagulopathy. The ERC also emphasizes the need for close reassessment after each bolus using clinical and biochemical parameters (169).

Similarly, the *National Institute for Health and Care Excellence (NICE)* guidelines recommend the use of glucose-free crystalloids containing sodium concentrations between 131 and 154 mmol/L. For children and young people requiring intravenous resuscitation, an initial bolus of 10 mL/kg over less than 10 min is advised. Special caution is warranted in patients with underlying cardiac or renal dysfunction, in whom lower volumes may be required. In term neonates, a bolus of 10–20 mL/kg is considered appropriate, tailored to clinical status and comorbidities (160).

## 7 Conclusion

Modern anesthesiology research shows that the avoidance of fluid-related complications require that the anesthetist possesses unique knowledge about human physiology in health and disease, fluid turnover, and how to titrate the fluid administration according to hemodynamic data. Despite the clear influence of the type and volume of fluid infused on postoperative outcomes after major surgery, high variability persists in daily practice, suggesting that fluid therapy is still too much a matter of personal experience and local practice. Importantly, the principles for fluid therapy cannot be uncritically extrapolated from adults to children. It is of utmost importance to gain an understanding of the physiological differences in children to avoid complications and to reduce the morbidity and mortality associated with surgery.
